# Estimating the impact of direct acting antiviral therapy on the prevalence of hepatitis C virus infection using phylogenetics

**DOI:** 10.1016/j.virusres.2025.199566

**Published:** 2025-03-26

**Authors:** Hossain M.S. Sazzad, Hui Li, Behzad Hajarizadeh, Bethany A. Horsburgh, Jason Grebely, Gregory J. Dore, Rowena A. Bull, Andrew R. Lloyd, Chaturaka Rodrigo

**Affiliations:** a Kirby Institute, UNSW, 2052 Sydney, NSW, Australia; b School of Biomedical Sciences, UNSW 2052, NSW, Australia

**Keywords:** Hepatitis C virus, Bayesian evolutionary analysis, People who inject drugs, Australia, Prisons

## Abstract

**Introduction:**

Australia has provided unrestricted subsidized access to direct-acting antiviral (DAA) treatment for hepatitis C virus (HCV) infection since 2016. Epidemiological surveillance estimates suggest prevalence of chronic HCV infection has declined since 2016, but these estimates are not separated by genotype and may not capture ‘hidden’ infected populations, notably the most marginalized groups affected, including people who inject drugs and people in prison. This study used phylogenetics to assess whether epidemiological estimates of declining HCV prevalence in the prisons of New South Wales, Australia due to DAA scale up could be reproduced.

**Method:**

Near-full-length 280 HCV consensus sequences (GT1a: *n* = 140, GT3a: *n* = 140) sampled between 2006 – 2019 from two prison-based cohort studies in NSW were used for phylogenetic estimates. These included 110 acute infection sequences (GT1a: *n* = 48, GT3a: *n* = 62) which were considered in a separate sensitivity analysis given the differences in virus mutation rates in acute and chronic infection. Changes in the effective population size of infected people for each genotype were explored with BEAST software suite (v1.10) using a coalescent Bayesian skyline approach.

**Results:**

Both the main and sensitivity analyses for GT3a showed a reduction in the effective population size with the latter showing a 36 % decline between 2011–2019 which is more concordant with the decline estimated from non-phylogenetic methods. A decline of similar magnitude was not demonstrated for GT1a. Overall, the analyses using acute infection sequences only were closer to the trends of independent epidemiological estimates.

**Conclusions:**

An adequately powered Bayesian evolutionary analysis using acute stage infection sequences may reproduce the decline in HCV infections observed by traditional epidemiological methods during DAA scale up.

## Highlights

• Phylogenetic models can complement traditional infectious diseases surveillance.
• Antivirals against hepatitis C infection became free-of-charge in Australia in 2016.
• Epidemiological surveillance found decline in Australian HCV infections since then.
• Phylogenetic modelling has limitations in replicating the same.
• The mutation rate differences in acute and chronic infection affects phylogenetic inferences.

## Graphical abstract

**Figure:**
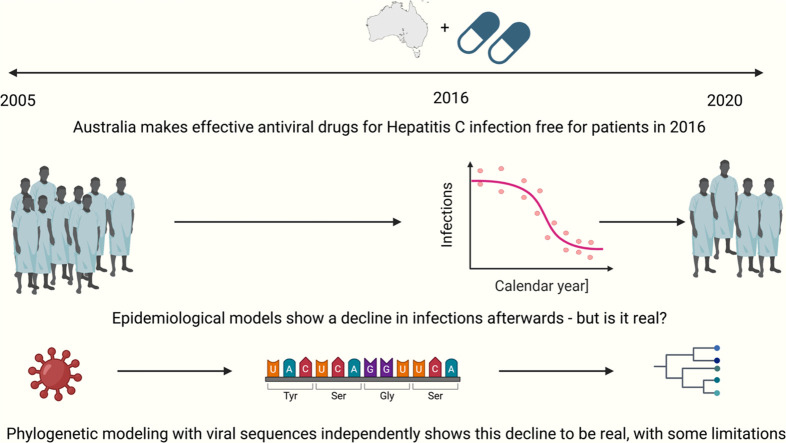

## 1 Introduction

Direct-acting antiviral (DAA) therapies have revolutionized the management of hepatitis C virus (HCV) infections since their introduction in 2011–2012, now achieving over 90 % cure for all genotypes with short course oral therapy (8–12 weeks). In 2016, the World Health Organization set targets for elimination of HCV as a public health threat by 2030, including three specific targets: to provide DAA treatment to 80 % of those with chronic HCV infection; to reduce incident HCV infections by 80 %; and to reduce liver-related mortality by 65 %. As of 2021, only a handful of the 194 signatory countries worldwide were on track to achieve these goals by 2030 (Polaris Observatory HCV Collaborators, 2022), reflecting the major barriers of the cost of DAAs (and uneven reimbursement rates and subsidies across high- and low-income countries) (Marshall et al., 2024), limitations in health infrastructure for diagnosis and treatment, and the marginalized nature of key populations needing treatment access (Marshall et al., 2024). Monitoring progress towards HCV elimination is a key element of implementation of the WHO strategy. This is traditionally achieved using epidemiological surveillance data to inform estimates of the numbers of prevalent and incident infections in the population. However, as HCV infection is largely asymptomatic in both acute and chronic phases, and epidemiological surveillance studies may not adequately sample hidden populations (People who inject drugs - PWID, people in prisons, people experiencing homelessness).

Australia has had universal access to testing and highly subsidized unrestricted access to DAA therapy under the Pharmaceutical Benefits Scheme (PBS) since 2016 (Hajarizadeh et al., 2018). The number of people living with HCV in Australia at end 2015, prior to the beginning of the DAA roll-out, was estimated at 162,590 (King et al., 2022; Kwon et al., 2019, 2021). This modelled estimate used cumulative laboratory notifications, estimated spontaneous clearance, mortality, and migration rates, and included an estimate for the percentage of people undiagnosed. In addition, the modelled mortality rates were calibrated to match empirical data from the NSW linkage study to best reflect the number of cases of decompensated cirrhosis, hepatocellular carcinoma, and liver‐related death. The resulting model estimates are compared to available measured data to ensure they are as accurate as possible. This process is repeated annually and can result in changes to the model estimates from year to year due to the availability of new data and information for re-calibration (Kirby Institute (UNSW Sydney), 2017; Burnet Institute and Kirby Institute, 2020; Papaluca et al., 2019). These data sources indicate a decline in both chronic HCV infection prevalence and in new HCV infections since the introduction of DAA treatment in Australia, but the data sources have limitations and require further validation.

Bayesian evolutionary analysis of viral sequence diversity has been successfully used to estimate the effective population sizes of infected people, within the limitations of the assumptions of its underpinning models (e.g., minimal or no recombination of viral sequences is assumed for coalescent models) (Drummond and Rambaut, 2007; Drummond et al., 2005, 2012; Golemba et al., 2010). Although these analyses are based on modelled populations, the trends of change over time in the estimated relative size of the infected populations have previously correlated well with observed epidemiological data (Rodrigo et al., 2016). These sequence-based models provide an alternative way to understand trends in the spread or control of an infection within a population and are especially useful when epidemiological estimates are uncertain. While neither method is perfect, the molecular and traditional epidemiological approaches often complement each other. Previously we used Bayesian skyline reconstruction of effective population sizes of HCV infected people in Australia using timed phylogenies of circulating viral sequences to show that both HCV GT1a and GT3a epidemics (the most common HCV genotypes found globally and in Australia) emerged in the 1920s and 1950s respectively, underwent a period of exponential growth until the 1970s and then stabilised up until 2012 (Rodrigo et al., 2016). In that analysis, sequences were not available from after 2012 to model changes in the effective population size after DAA roll-out. In this study Bayesian evolutionary analysis of HCV sequences generated from two prison-based cohorts before and during DAA roll-out was explored to see if this phylogenetic method could reproduce the epidemiologically estimated decline (Dore and Hajarizadeh, 2018; Kirby Institute and Australia, 2021; Justice Health, 2017) in the number of HCV infected individuals in the prisons of New South Wales, Australia.

## 2 Methods

### 2.1 Clinical samples and sequences

The clinical samples for this study were sourced from the Hepatitis C Incidence and Transmission Study in prisons (HITS-p) (Cunningham et al., 2018), and the Surveillance and Treatment of Prisoners with hepatitis C (SToP-C) study (Hajarizadeh et al., 2021a). The HITS-p cohort which provided sequences from the pre-DAA era prospectively recruited from 37 prisons in New South Wales (NSW) between 2005 and 2014, enrolling PWID who were incarcerated and who were HCV uninfected at baseline (Bretana et al., 2015; Cunningham et al., 2017; Luciani et al., 2014). The participants were screened at baseline for both anti-HCV antibodies and HCV RNA and followed-up at 6 monthly intervals with repeat testing. Plasma samples were stored from all participants who became infected during regular follow up. The SToP-C study evaluated DAA treatment scale-up as a prevention strategy within four NSW prisons and recruited adult inmates regardless of risk behaviour and HCV status between late-2014 and 2019. HCV-infected individuals were treated with DAAs, and uninfected individuals were followed at 6 monthly intervals for infection (Hajarizadeh et al., 2021a). Treated individuals were also followed up for re-infection. Pre-treatment plasma samples were preserved from all infected patients. The details of both these cohorts are published elsewhere (Cunningham et al., 2018; Hajarizadeh et al., 2021a) and when taken together, these samples provided a unique opportunity to observe how viral sequence diversity changed over time from the pre-DAA era (2005 – 2015) into the DAA era (2016 - 2019).

Preserved plasma samples from both cohorts were used for full viral genome extraction and amplification as previously described by Bull et al. (Bull et al., 2016), provided that the sample was infected with HCV GT1a or GT3a, and had a viral load of at least 1000 IU/ml. While both acute and chronic infection sequences were included in the main analysis, Given the different rates of viral evolution between acute and chronic infections (Bull et al., 2011) and to compare with a similar analysis published previously with better accuracy (Rodrigo et al., 2016), a sensitivity analysis was performed with only the acute infection sequences. A viral sequence identified from a sample collected within 180 days since the estimated date of infection was designated as an acute infection sequence, a cut-off used in previously published research on HCV phylogenetics (Rodrigo et al., 2016). The estimated date of infection was calculated as the mid-point between the last HCV antibody negative date and the first HCV antibody positive date (Cunningham et al., 2018), or as 6 weeks prior to the first date of HCV RNA positivity in individuals who had no anti-HCV antibodies at the first sampling timepoint (Cox et al., 2005). Only one plasma sample was used for viral sequence extraction per participant per infection episode, and when multiple samples were available per participant, the earliest available sample was prioritized.

The viral genomic amplicons were size selected with the Monarch DNA Gel Extraction Kit (catalogue number: T1020S, New England Biolabs, USA), cleaned with magnetic beads (Agencourt AMPure XP, Beckman Coulter, USA, Cat: A63881) and sequenced with Oxford Nanopore Technology on a GridION sequencer using a FLO-MIN107 v9.5 flow cell with ligation barcoding (ONT ligation sequencing kit SQK-LSK109). Signal-level data from the sequencer was base-called, filtered for high-quality reads (mean Q-score >7) and demultiplexed with Guppy (version 2.3.5 and 3.0.3, http://staff.aist.go.jp/yutaka.ueno/guppy/) and further processed with the Nanopolish algorithm as described previously to generate an output in Fastq format (Riaz et al., 2021).

The output from the above step was imported into Geneious prime software (Dotmatics, Boston, USA), and aligned iteratively in two steps - firstly against a generic genotype-specific reference, and then against the autologous reference generated from the first step. The final consensus sequences were used to make genotype specific, near-full-length sequence alignments with the MUSCLE algorithm (Edgar, 2004). Each sequence was tagged with the date of sampling, converted to a decimal notation (e.g., June 30, 2012, was noted as 2012.5)

### 2.2 Bayesian evolutionary analysis

This analysis of viral evolution was undertaken with the BEAST software suite (v.1.10) (Drummond and Bouckaert, 2015), for each genotype (1a and 3a) separately. The alignments from the previous steps were exported in nexus format to be used as the input for BEAUti (version 1.10) software (Drummond and Rambaut, 2007; Drummond et al., 2012) to generate the input .xml file required for BEAST v1.10. To balance having an adequate sampling density against the higher computational needs with a larger input dataset, the size of each genotype specific alignment was limited to 140 sequences (approximately 10 samples per year across the 14-year sampling window from 2006–2019). To select the best fitting clock model (strict or lognormal relaxed) and substitution model (Tamura-Nei 93 or General time reversible or Hasegawa-Kishono-Yano model), the alignments were tested in a 3 × 2 experimental design by path and stepping-stone sampling method(Lartillot and Philippe, 2006; Xie et al., 2011) which showed that none of the models performed significantly better than the other. Thus to maintain consistency of models used with our previous publication in 2016 limited to pre-DAA era sequences(Rodrigo et al., 2016), the analyses were run in BEAST using each of: a lognormal relaxed clock model, a generalized time reversible nucleotide substitution model, and a coalescent-Bayesian skyline population model. Only the coding region of the genomes were used. The Monte Carlo Markov Chain (MCMC) which estimates values for each parameter in the analysis was run with a sampling frequency of 1 per 10^6^ states. An effective sample size (ESS) >200 (per parameter) was considered as adequate sampling to indicate that the MCMC had reached an equilibrium. The MCMC continued for a length of 10^9^ states or until all parameters reached an equilibrium whichever was the longest. The output was visualized in Tracer software (version 1.7) (Rambaut et al., 2014). A Maximum Clade Credibility (MCC) phylogenetic tree was generated from the sampled tree states using TreeAnnotator (version 1.8.2, 10 % burn-in) tool (Drummond et al., 2012), and was visualized on Figtree software (version 1.4.2) https://github.com/rambaut/figtree/releases). All analyses were run on the CIPRES gateway (version 3.3, https://www.phylo.org/) using the high-performance BEAGLE library (Ayres et al., 2012) for computations. The temporal changes in the effective population size of people infected with HCV as predicted by the phylogenetic tree were visualized on a Bayesian skyline plot generated by the Tracer software.

### 2.3 Epidemiological data

Epidemiological data to compare with the trends observed in the Bayesian skyline projections were obtained from the SToP-C study(Hajarizadeh et al., 2021a), National Prison Entrants Blood Borne Virus Surveys (2004, 2007, 2010, 2013, 2015) (Kirby Institute (UNSW Sydney), 2017), Justice Health and Forensic Mental Health Network (JH&FMHN) NSW Survey (2015) (Justice Health, 2017), and Australian Needle and Syringe Program Survey (ANSPS) data (2004 – 2020) (Iversen and Maher, 2015; Kirby Institute and Australia, 2021). The latter is not a prison-based but as a national survey it provides an insight into the overall HCV infection trends in Australia.

## 3 Results

From both cohorts 693 eligible viraemic samples were identified (GT1a – *n* = 344, GT3a – *n* = 349), and of these 181 (26.1 %) were acute infections (GT1a – *n* = 95, GT3a – *n* = 86). From all samples 537 (77.5 %) near full length genomes (>8950 nucleotides) were successfully generated (GT1a – *n* = 241, GT3a – *n* = 296). Of these 110 (110/181, 60.8 %) were acute infection sequences (GT1a – *n* = 48, GT3a – *n* = 62). For the remainder, sequences could not be generated either due to sample unavailability, poor RNA quality or the final consensus sequence not passing the quality control requirement (>2 % of nucleotides in the consensus sequence having a margin of error >0.0001 for base calling). Two genotype specific alignments were (GT1a – *n* = 140, GT3a – *n* = 140) separately subjected to Bayesian evolutionary analysis as described in Methods. These alignments contained all acute infection sequences and randomly selected chronic infection sequences up to a pre-determined cap of 140 sequences. The year of sampling of included sequences is shown in Supplementary Table 1.Both analyses successfully equilibrated with an ESS> 200 for all parameters after a MCMC chain length of 10^9^ states. The results for some key parameters from these analyses (posteriors and 95 % high posterior density interval) are summarized in Table 1 and in the supplementary datafile 1.

**Table 1:** Summary of results of the evolutionary analysis of genotype 1a and 3a sequences using BEAST v1.10.

Parameter*	1a – acute and chronic (*n* = 140)	1a – acute (*n* = 48)	3a – acute and chronic (*n* = 140)	3a – acute (*n* = 62)
Mean tMRCA^†^ in years	107.3	109.9	110.1	66.6
Mean tMRCA in calendar year	1912.5	1909.9	1909.8	1953.3
tMRCA – 95 % high posterior density	1859 – 1956	1804.9 – 1978.1	1844.4 – 1958.6	1920.6– 1978.5
Mean substitution rate^‡^	8.5	8.79	6.7	10.0
Substitution rate – 95 % high posterior density^‡^	5.18 – 12.0	2.5 – 15.2	3.3 – 10.0	5.6 – 14.8

⁎ All parameters in this table (including posterior, prior, likelihood, tree likelihood – not shown here) reached equilibration with an effective sample size > 200 after a MCMC chain length of 10^9^ states sampled every 10^5^th state. †tMRCA – Time to most recent common ancestor, ‡substitution rate (× 10^–4^ substitutions per site per year).

**Fig. 1:**
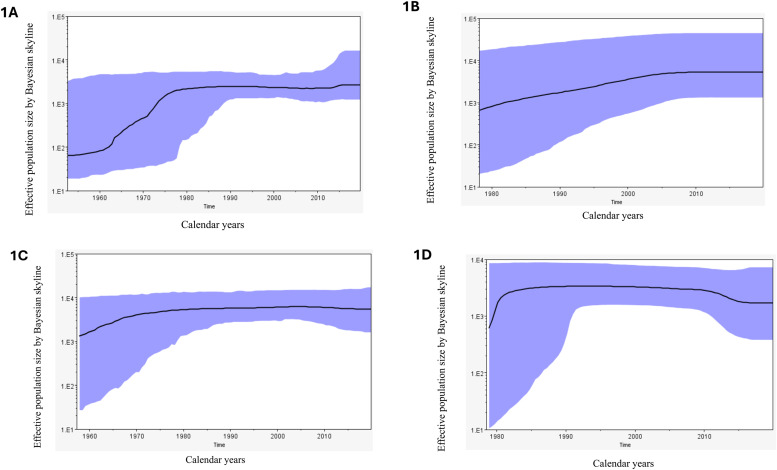
Bayesian skyline reconstruction of the effective population size of infected people. The X-axis indicates time in calendar years and the Y-axis shows the effective population size in log scale. The black line indicates the median effective population size while the shaded area refers to the 95 % high posterior density interval. 1A – GT1a main analysis, 1B – GT1a acute infection only analysis, 1C – GT3a main analysis, 1D – GT3a acute infection only analysis.

For GT1a analysis, the time to the most recent common ancestor (tMRCA) of sampled sequences existed around 191 2 (95 % HPD: 1859 – 1956) and the mean evolutionary rate was 7.58 × 10^–4^ substitutions/site/year (95 % HPD: 4.4 – 10.8 × 10^–4^). The Bayesian skyline projection which maps the effective population size (a modelled population of those living with infection) in the y axis against calendar years on the x axis (Fig. 1, Supplementary datafile 1) shows that the number of infections (median estimate) were stable or slightly declined between 2000 – 2012 (2 % decline overall) followed by a 18 % rise between 2012 – 2016 and then remained static between 2016 - 2019. When the analysis was repeated with acute infection sequences only, the tMRCA and evolutionary rate estimates changed slightly but with a wider 95 % HPD (Table 1). In the corresponding Bayesian skyline projection, the median number of infections remained static between 2010 – 2019.

For GT3a sequences, the tMRCA was estimated to have been around 190 8 (95 % HPD: 1841 – 1957) and the mean evolutionary rate was approximately 6.67 × 10^–4^ substitutions/site/year (95 % HPD: 3.3 – 10.2 × 10^–4^). The Bayesian skyline projection showed the effective population size (median estimate) peaked in 2004 before gradually declining by 13.2 % by 2019 (Fig. 1, Supplementary datafile 1). When the acute infections only were considered the tMRCA was more recent and a faster evolutionary rate was noted (Table 1). The skyline projection also showed a sharp decline of 36 % of the effective population size between 2011–2017, after which the numbers remained static between 2018–2019 (Fig. 1, Supplementary datafile 1). The consensus sequences used in all analyses are provided as supplementary datafiles 2- 5.

**Fig. 2:**
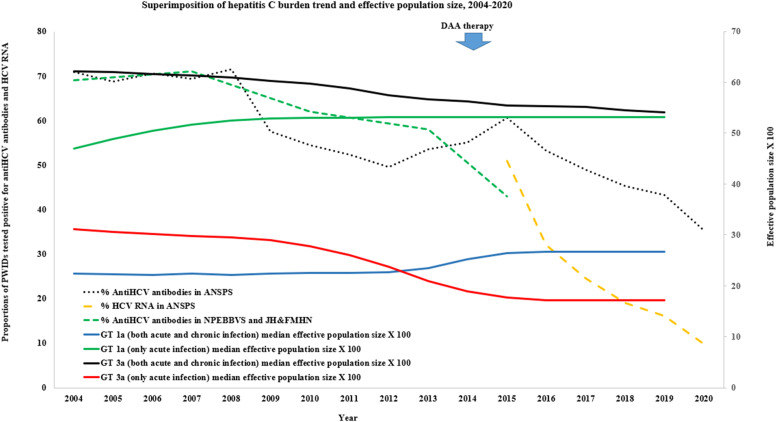
A superimposition of the HCV GT1a, GT3a trends derived from the Bayesian skyline projections on top of several epidemiological projections (Note – ANSPS: Australian Needle and Syringe Program Survey is a Nationally representative, non-prison-based survey, JH&FMHN: Justice Health and Forensic Mental Health Network survey in NSW, NPEBBVS: National Prison Entrants Blood Borne Virus Surveys – NSW data).

We superimposed the trends derived from the Bayesian skyline projections on top of several epidemiological projections (Fig. 2). The epidemiological projections could not be split by genotype as this information was not reported. Only the trends of acute infection GT3a analysis were largely concordant with the trends in the epidemiological estimates (Fig. 2). The SToP-C – study showed an overall 48 % decrease in HCV incidence ((8·31 per 100 person-years to 4·35 per 100 person-years) and a comparable fall in chronic HCV prevalence (19 % to 10 %) due treatment scale-up in NSW prisons. In other surveys, the Australian Needle and Syringe Program Survey (ANSPS) data for NSW shows a 26 % decline in the prevalence of HCV antibodies and a 41 % decline in the HCV RNA prevalence between 2015–2020. Similarly, the HCV antibody prevalence among people who inject drugs observed by the National Prison Entrants Blood Borne Virus surveys in NSW show a 15 % decline between 2013–2015. However, a similar decline was not seen in our analysis for GT1a (or for GT3a when acute and chronic infection sequences were mixed).

## 4 Discussion

This Bayesian evolutionary analysis based on the diversity of HCV sequences sampled from NSW prisons between 2006–2019, showed a significant decline of the effective population size of HCV infections concordant with the epidemiological estimates only for GT3a, when the analysis was limited to acute infection sequences only.

Within-host evolution of HCV is complex due to the generally persistent (chronic) nature of infection (compared to acute viral infections like influenza) as the time elapsed since infection (which is typically unknown and prolonged) influences the mutation rate. Also, the evolutionary rate in acute infection is higher than in chronic infection as the virus adapts to immune system of the new host and typically undergoes two genetic bottleneck events (transmitted-founder effect) within the first 100 days of infection (Bull et al., 2011). Also, approximately 23 – 25 % of all HCV infections spontaneously resolve within the acute stage of infection (i.e. the first 6 months, more common in GT1 infections) (Grebely et al., 2014). The evolutionary dynamics (and mutation rates) of infections that eventually resolve may be different than those becoming established as chronic infection. Therefore, the differences in virus evolution in acute and chronic infections may impact on the phylogenetic inferences and in our opinion using acute infection sequences only may be more accurate to model the changes in HCV incidence (similar to viral infections without a chronic stage like influenza). However, HCV infection is clinically asymptomatic until the late stages of disease which may take years or decades to manifest and it is therefore very difficult to isolate and sequence acute infection sequences. This study had the advantage of using samples from two prospective studies in NSW prisons that allowed identification of acute infections. However, the viral load in the acute infection time window (the first 6 months of infection) is often relatively low, limiting the success rate in generating near-full length HCV sequences. This is further complicated by the preferential clearance of GT1 infections during this time window. However, the success rate in extracting and sequencing the near-full-length HCV genome in this analysis was comparable to that reported by other studies that used the same assay (Bull et al., 2016; Rodrigo et al., 2017).

The historical trends in the effective population sizes, time to most recent common ancestor and the evolutionary rates observed in this study with acute infection sequences are comparable to the previously published study of Australian acute infection sequences (Rodrigo et al., 2016). This study goes one step further to extend the time window of analysis to include the critical time window of 2011–2019 during which DAA therapy was made available in Australia subsidized under the pharmaceutical benefits scheme (2016-). Based on estimated projections from epidemiological surveys and national prescription data, there was a significant reduction in the number of people living with HCV in Australia in the initial years of the roll out (Alavi et al., 2019; Dore and Hajarizadeh, 2018; Kirby Institute and Burnet Institute, 2019; Kwon et al., 2019; Papaluca et al., 2019). However, recently the number of new DAA treatment initiations per annum has fallen as most “easy to engage” patients have been treated. This includes individuals from marginalized populations, as treatment coverage among people who inject drugs and people in prisons is equivalent to the broader HCV population (Bah et al., 2024; Valerio et al., 2021). As DAA treatment and cure does not prevent re-infection, and the notification systems do not reliably capture such events, data from SToP-C study suggests that for those incarcerated both the rate of primary HCV infection (39 per 100 person-years during pre-DAA era to 14 per 100 person-years after the DAA scale-up), and the rate of HCV reinfection reduced (decreasing from 15 to 9 per 100 person-years during the same periods). In addition during the course of the study chronic HCV prevalence fell from 19 % to 10 % amongst the incarcerated population (Hajarizadeh et al., 2021b).

This analysis has several limitations. Firstly, there was uncertainty regarding the precise date of infection with a margin of error of up to −6 months, and sampling bias towards a high viral load and GT3a infections. Secondly, the period decline in Bayesian estimates (2011 – 2017) do not show an exact overlap with the decline of epidemiological survey estimates (2016 – 2020). Given the large high posterior densities of the Bayesian estimates, it is unrealistic to expect sufficient precision from the phylogenetic analysis for an exact overlap. On the other hand, there are also uncertainties in the epidemiological datasets used for comparison as well given that some surveys were not done at regular intervals. Thirdly, this study only sampled from the NSW prisons where the risk of transmission may be higher than outside of prisons. Therefore, the findings cannot be generalized to all people living with HCV in NSW. The average length of stay of inmates in the NSW prisons is approximately 8 months indicating a high rate of movement in and out of prisons (NSW Bureau of Crime Statistics and Research, 2021) and mixing with the outside community. However, the prison population is dissimilar to the wider NSW community in that males, Indigenous people, some ethnic minorities and younger age groups are over-represented (NSW Bureau of Crime Statistics and Research, 2023). Further the incidence rates of new HCV infections in the HITS-p study used in the current analysis and the largely contemporaneous counterpart epidemiological study (HITS-c) were 11.4 versus 7.9 per 100 person years respectively (Cunningham et al., 2018; White et al., 2014). It is likely therefore that new infections occur at a higher rate in the prisons (hence allowing faster viral evolution) reflecting the higher risk environment for injecting drug use within the prisons. Fourthly, the trends of infection over time in the phylogenetic analyses are explained based on the median estimate but the high posterior density of each time-point estimate in the Bayesian skyline is wide especially for the acute infection sequence analyses as shown in the Supplementary datafile 1. Finally, the coalescent model assumes random sampling from the population. An assumption that may be violated in a “closed community” with a high transmission risk like prisons by including closely related transmissions than that can be explained by chance (e.g., including one or more transmission clusters). This will lead to more recent coalescent events in one subtree, spuriously underestimating the effective population size near the “present time”. This effect is more so for acute infection sequence analyses where the sample size was smaller.

None of the above limitations were genotype specific (except for the sampling bias) but the analysis for GT1a acute infection sequences failed to show a decline as was seen for the equivalent GT3a analysis. The common trends observed by multiple independent surveys and the conclusions of the SToP-C study derived from epidemiological analyses all suggest that the incidence and prevalence of HCV infections have declined in NSW prisons since the roll-out of DAAs. Since most HCV infections in NSW are of GT1a and GT3a (Walker et al., 2016), and because GT3a infections were somewhat less responsive to the early DAAs such as sofosbuvir/ledipasvir (available in Australia from 2016), and as both genotypes are effectively cured with newer pangenotypic DAAs (available from 2017 in Australia) it is unlikely that only GT3a infections would have selectively declined with the DAA roll-out. Hence, the failure to observe a decline for GT1a infections in this analysis is most likely to be due to an underpowered analysis as the number of acute infections GT1a sequences available was smaller. Another explanation is that the GT1a infections were more likely to be spontaneously cleared without DAA treatment during the acute infection time window, and hence transmission events were inadequately captured and sequenced (Grebely et al., 2014).

## 5 Conclusion

Bayesian evolutionary analyses may be a useful tool to complement epidemiological surveillance data for RNA virus infections in general, but its use in HCV to reproduce recent anticipated changes in incidence after DAA roll-out must be done with caution. The different mutation rates in acute and chronic stages of HCV infection may influence the accuracy of the phylogenetic inferences and it is recommended to use acute infection sequences whenever possible. Admittedly given the asymptomatic nature of acute HCV infection and genotype-specific differences in spontaneous clearance of infection, obtaining sufficient numbers of acute infection sequences can be challenging for similar analyses especially when infections are dominated by GT1a. The advances in HCV virus full length genome amplification assays, and increasing availability and sensitivity of low-cost sequencing (e.g., Oxford Nanopore Technology) would hopefully increase the yield and availability of acute infection HCV sequences.

## CRediT authorship contribution statement

**Hossain M.S. Sazzad:** Writing – original draft, Methodology, Investigation, Formal analysis, Data curation. **Hui Li:** Validation, Methodology, Investigation. **Behzad Hajarizadeh:** Writing – review & editing, Resources, Methodology. **Bethany A. Horsburgh:** Writing – review & editing, Validation, Investigation. **Jason Grebely:** Writing – review & editing, Resources, Methodology, Funding acquisition. **Gregory J. Dore:** Writing – review & editing, Resources, Investigation, Funding acquisition. **Rowena A. Bull:** Writing – review & editing, Resources, Methodology, Investigation. **Andrew R. Lloyd:** Writing – review & editing, Supervision, Resources, Methodology, Investigation, Funding acquisition, Conceptualization. **Chaturaka Rodrigo:** Writing – original draft, Supervision, Project administration, Methodology, Investigation, Funding acquisition, Formal analysis, Data curation, Conceptualization.

## Declaration of competing interest

The authors declare the following financial interests/personal relationships which may be considered as potential competing interests:

Chaturaka Rodrigo reports financial support was provided by National Health and Medical Research Council. Hossain Sazzad reports financial support was provided by University of New South Wales. Gregory Dore reports financial support was provided by National Health and Medical Research Council. Andrew Lloyd reports financial support was provided by National Health and Medical Research Council. Rowena Bull reports financial support was provided by National Health and Medical Research Council. Jason Grebely reports financial support was provided by National Health and Medical Research Council. If there are other authors, they declare that they have no known competing financial interests or personal relationships that could have appeared to influence the work reported in this paper.

## Data Availability

All data attached as supplementary material.

## Appendix Supplementary materials

Image, application 1

Image, application 2
