# Supplementary material for: Estimating the impact of direct acting antiviral therapy on the prevalence of hepatitis C virus infection using phylogenetics
Source: Virus Res. 2025 Mar 26;355:199566. doi: 10.1016/j.virusres.2025.199566 (PMC11999633; doi:10.1016/j.virusres.2025.199566)
Supplement: Supplementary file 1 [file mmc1.docx]

Supplementary Table 1. Frequency of available acute infection HCV sequences by calendar year and genotype

| Calendar Year | GT1a | | GT3a | |
| --- | --- | --- | --- | --- |
|  | Acute | Chronic | Acute | Chronic |
| 2006 | 0 | 5 | 1 | 12 |
| 2007 | 1 | 5 | 2 | 8 |
| 2008 | 5 | 6 | 4 | 3 |
| 2009 | 2 | 1 | 5 | 2 |
| 2010 | 1 | 0 | 3 | 0 |
| 2011 | 3 | 0 | 4 | 0 |
| 2012 | 6 | 0 | 6 | 0 |
| 2013 | 2 | 0 | 5 | 0 |
| 2014 | 3 | 0 | 3 | 5 |
| 2015 | 0 | 8 | 3 | 4 |
| 2016 | 6 | 19 | 1 | 8 |
| 2017 | 5 | 16 | 10 | 11 |
| 2018 | 4 | 21 | 9 | 18 |
| 2019 | 10 | 11 | 6 | 7 |
| Total | 48 | 92 | 62 | 78 |
